# Self-sustained green neuromorphic interfaces

**DOI:** 10.1038/s41467-021-23744-2

**Published:** 2021-06-07

**Authors:** Tianda Fu, Xiaomeng Liu, Shuai Fu, Trevor Woodard, Hongyan Gao, Derek R. Lovley, Jun Yao

**Affiliations:** 1 Department of Electrical and Computer Engineering, University of Massachusetts, Amherst, MA USA; 2 Department of Microbiology, University of Massachusetts, Amherst, MA USA; 3 Institute for Applied Life Sciences (IALS), University of Massachusetts, Amherst, MA USA; 4 Department of Biomedical Engineering, University of Massachusetts, Amherst, MA USA

**Keywords:** Electrical and electronic engineering, Biomaterials - proteins

## Abstract

Incorporating neuromorphic electronics in bioelectronic interfaces can provide intelligent responsiveness to environments. However, the signal mismatch between the environmental stimuli and driving amplitude in neuromorphic devices has limited the functional versatility and energy sustainability. Here we demonstrate multifunctional, self-sustained neuromorphic interfaces by achieving signal matching at the biological level. The advances rely on the unique properties of microbially produced protein nanowires, which enable both bio-amplitude (e.g., <100 mV) signal processing and energy harvesting from ambient humidity. Integrating protein nanowire-based sensors, energy devices and memristors of bio-amplitude functions yields flexible, self-powered neuromorphic interfaces that can intelligently interpret biologically relevant stimuli for smart responses. These features, coupled with the fact that protein nanowires are a green biomaterial of potential diverse functionalities, take the interfaces a step closer to biological integration.

## Abstract (web summary)

For bio-inspired neuromorphic interfaces to emulate biological signal processing and self-sustainability, the mismatch between sensing and computing signals must be addressed. Here, the authors report sensor-driven, integrated neuromorphic systems with signal matching at the biological level.

## Introduction

Biologically inspired electronic interfaces and microsystems take advantage of unique properties and functions that are optimized in the natural world^1–4^. For example, memristors that emulate biological signal processing can improve computing efficiency^5,6^. Neuromorphic interfaces made from memristors emulate the signal processing of neural components in order to efficiently and rapidly process sensing signals from electrical, optical, mechanical, and thermal stimuli^7–11^. However, substantial differences between previously described neuromorphic interfaces and biosystems remain. Biological receptors elicit sensory signals (e.g., action potentials) that directly drive bio-computation. In contrast, many sensors require an external energy input^7,8^. Recent works demonstrated neuromorphic responses driven by active sensors made from energy devices (e.g., mechanic and thermoelectric generators)^10,11^. However, these functional demonstrations were limited to a specific stimulus and off-chip connection not readily applicable to the compact and flexible integrated interface.

The key challenge to developing sensor-driven, integrated neuromorphic interfaces is the inherent amplitude mismatch between the sensing and computing signals. The general approach is to increase the sensing signal through the careful selection of sensor structure and stimulus^10,11^. However, this approach often leads to requirements in the sensor’s form factor and/or working environment that do not accommodate a compact or flexible integration. Furthermore, some environmental or physiological signals are inherently limited in amplitude^12,13^. In contrast, biosystems use a different approach by reducing the computing signal (e.g., 50–120 mV action potentials) close to thermodynamic limit^13^, enabling bio-computation responding to a much broader range of environmental stimuli. The ultralow-amplitude signal processing underlies the unified sensory and computing functions in biosystems, which manifests to be the key to advancing integrated neuromorphic interfaces.

We demonstrate fully sensor-driven, integrated neuromorphic systems by achieving signal match at the biological level. The systems are built on three recently recognized properties of protein nanowires produced from the microbe Geobacter sulfurreducens^14–16^. First, memristors made from protein nanowires can be driven by sub-100 mV signals^17^, enabling bio-amplitude neuromorphic circuits and signal processing. Second, devices fabricated with protein nanowires can harvest electric energy from environmental humidity^18^, sustaining signal and energy to computing. Third, protein nanowires can function as the sensing component in electronic sensors^19^. The bio-composition in the devices also represents an exploration for “green” electronics integrated from biomaterials that offer renewability, biocompatibility, and eco-friendliness^16^. The protein nanowires also enable easy reconfigurable functions for adaptive microsystems. Together, these properties in bio-integration, self-sustainability, and reconfigurability feature a step further in advancing bio-emulated interfaces and microsystems.

## Results

### Green electronic components

**Fig. 1:**
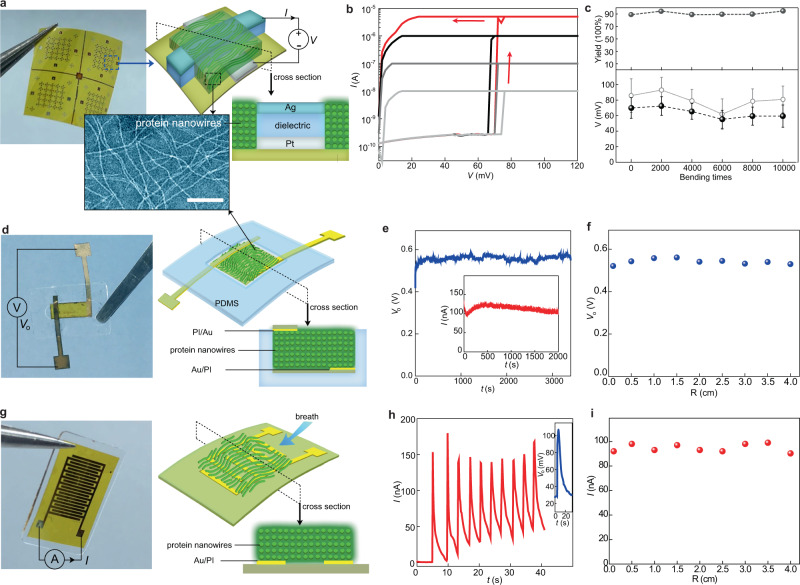
Flexible protein nanowire devices. **a** (Left) Fabricated protein nanowire memristor arrays on a polyimide (PI) substrate and (right) the schematics of the device structure. (Bottom) Transmission electron microscope (TEM) image of protein nanowires. Note that the actual nanowire density is much higher in assembled film. Scale bar, 100 nm. **b** Switching *I–V* curves from a memristor with the current compliance (*I*_CC_) set at different values from 5 µA to 10 nA. **c** Device yield (top), threshold switching voltage (black, bottom), and forming voltage (gray, bottom) in protein nanowire memristors subjected to various bending times. The error bars represent the standard deviation (s.d.). **d** (Left) A fabricated protein nanowire sensor with a vertical structure as shown in schematics (right). **e** Open-circuit voltage (*V*_o_) and short-circuit current (inset) from the vertical protein nanowire sensor in the ambient environment (RH ~50%). **f** Output voltage *V*_o_ from the vertical protein nanowire sensor at different bending radius of 4–0.1 cm. **g** (Left) A fabricated protein nanowire sensor with a planar structure as shown in schematics (right). **h** Current and voltage (inset) signals generated in the planar protein nanowire sensor elicited by breathing. **i** Breathing-induced peak current in a planar protein nanowire sensor at a different bending radius of 4–0.1 cm.

We first examined the key components for the integration in a bio-realistic environment that often features softness and curvature. Protein nanowire memristors were fabricated on a flexible substrate. The device was a vertical structure with an insulating layer (20 nm SiO_2_) sandwiched between a top (150 nm Ag) and a bottom electrode (17 nm Pt). This structure was embedded in a thin layer (~500 nm) of protein nanowire film (Fig. 1a, Supplementary Fig. 1, “Methods” section). The device functioned at a power level comparable to biological neuron in terms of programming voltage and current. The state transitioned to low resistance at 70 ± 3 mV (±s.d.) and spontaneously relaxed to high resistance at close-to-zero bias (Fig. 1b). The switching was robust and repeatable (Supplementary Fig. 2a) and showed a robust endurance of 10^4^ cycles in pulsed programming^17^. The switching was independent of device size (Supplementary Fig. 2b), consistent with a filamentary switching mechanism^20,21^. Statistics from 117 devices show a consistent switching voltage (*V*_th_) of 65 ± 14 (±s.d.) (Supplementary Fig. 2c), which is within the range of biological action potentials^13^. *V*_th_ was achieved with a programming current (*I*_cc_) as low as 0.1–10 nA (Fig. 1b, Supplementary Fig. 2d), comparable with the membrane current during an action potential^13^. As a result, the protein nanowire memristor features the lowest programming power among the similar types of devices (Supplementary Table 1).

Devices without protein nanowires could not achieve bio-amplitude switching (Supplementary Fig. 3), pointing to the key role protein nanowires play in enabling the bio-amplitude switching. A recent study shows that low activation energy in vacancy migration can lead to close-amplitude switching based on a valance change mechanism^22^. The switching here is based on the mechanism of electrochemical metallization characterized by the observation of metal filaments^17^. The protein nanowires are uniquely designed for charge transfer in microbes^14–16^. Electrons derived from cellular metabolism can be donated through the protein nanowires, with the protein nanowires serving as points of nucleation and reduction of metals^23^. Ag^+^ ions can be readily reduced to Ag nanoparticles by G. sulfurreducens in the environment^24^, indicating that protein nanowires can facilitate Ag^+^/Ag redox. Cyclic voltammetry indeed showed that protein nanowires yielded a Ag^+^/Ag reduction potential lower than inorganic dielectric used in conventional memristors^17^. Protein nanowires thus may have facilitated Ag metallization during filament formation to reduce *V*_th_^17^. The molecular details of facilitation warrant further investigation.

Bending tests revealed stability in the memristors for wearable implementation (Supplementary Fig. 4). The devices maintained a high yield (~90%) and stable switching after being bent 10,000 times (top panel, Fig. 1c). The average *V*_th_ (*N* = 15) remained within a narrow distribution of 55–75 mV (bottom panel, Fig. 1c), with the forming voltage staying close to a forming-free value of 60–95 mV (gray circles). These key parameters show no deviation from devices on a flat substrate^17^. The mechanical flexibility in the memristors is attributed to (1) a thin device thickness (e.g., 500 nm) offering reduced bending stiffness and (2) an intrinsic flexibility expected in protein nanowires (e.g., 3 nm diameter)^14^.

We next evaluated active sensors made from protein nanowires. Two device configurations were used for different responses. The first one was a vertical structure with a protein nanowire film (*~*5 µm thick) sandwiched between a top and bottom Au electrode, which was further embedded between two polydimethylsiloxane (PDMS) layers for flexible integration (Fig. 1d and “Methods” section). The protein nanowire film builds up a vertical moisture gradient in the ambient environment for sustained electric outputs (Fig. 1e)^18^. Bending the device had negligible influence on its output (Fig. 1f). The second one was a planar structure with a protein nanowire film (~1 µm thick) deposited on a pair of interdigitated electrodes (Fig. 1g and “Methods” section). Due to in-plane symmetry, the device did not generate a voltage in the ambient environment (Supplementary Fig. 5). However, a quick change of local humidity as the result of breathing-induced non-uniform moisture adsorption^19^, resulting in an instant electric spike (Fig. 1h). Mechanical bending had a negligible effect on the performance (Fig. 1i).

The estimated steady and instant power outputs in the above vertical and planar devices (without energy optimization) were ~40 and 10 nW/cm^2^, respectively, which were more than an order of magnitude larger than the minimal switching power in the protein nanowire memristor (e.g., <1 nW, Fig. 1b). This offers the feasibility to drive neuromorphic circuits at a small sensor size (e.g., <1 cm^2^), a key to compact integration desired in wearable devices and microsystems. Importantly, power is not the only constraint. The field-driven mechanism in memristors also requires a voltage above a certain threshold^25,26^. As energy devices can have large internal resistance (e.g., comparable to the Off resistance in memristors)^18,27^, an output voltage comparable to the threshold is still insufficient due to the voltage-divider effect. Thus, some active sensors were able to drive synaptic modulation in typical memristors^28,29^, but may not drive somatic function (e.g., neuronal firing). Therefore, a bio-amplitude *V*_th_ is crucial in realizing sensor-driven neuromorphic functions. The vertical and planar protein nanowire devices can produce continuous (Fig. 1e) and spiking (Fig. 1h) signals, respectively, enabling different functional interfaces.

### Amplitude-driven neuromorphic interfaces

**Fig. 2:**
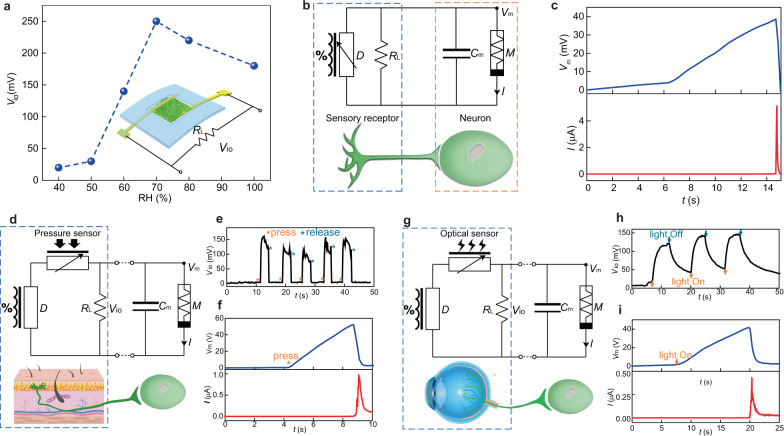
Multifunctional sensory neuromorphic interfaces. **a** Voltage output (*V*_io_) from a vertical protein nanowire sensor connected with a load resistor (*R*_L_) measured at different RH. **b** Circuit diagram of feeding *V*_io_ from the protein nanowire sensor (*D*) into an artificial neuron made from a protein nanowire memristor (*M*) and a capacitor (*C*_m_) by emulating an afferent circuit (bottom). **c** Evolution in the membrane potential (*V*_m_) and current (*I*) in the artificial neuron when placed the sensor on a sweating skin. **d** Circuit diagram of feeding *V*_io_ from a tactile sensory component (made from a protein nanowire device (*D*) and a resistive pressure sensor) into an artificial neuron by emulating a tactile afferent circuit (bottom). **e** Touch-induced *V*_io_ from the tactile sensory component. **f** Evolution in the membrane potential (*V*_m_) and current (*I*) in the artificial neuron by pressing the pressure sensor. **g** Circuit diagram of feeding *V*_io_ from an optical sensory component (made from a protein nanowire device (*D*) and an optical sensor) into an artificial neuron by emulating an optical afferent circuit (bottom). **h** Light-induced *V*_io_ from the optical sensory component. **i** Evolution in the membrane potential (*V*_m_) and current (*I*) in the artificial neuron by lighting the optical sensor.

We first demonstrated neuromorphic interfaces made from the vertical energy devices and memristors. The energy device was connected to a resistor (*R*_L_) in parallel to form a sensory component (Fig. 2a). The output voltage (*V*_io_) substantially increased from below 30 mV to above 100 mV when the relative humidity (RH) was changed from 50 to 60% (Fig. 2a). Note that the open-circuit voltage of the device (i.e., without *R*_L_) did not vary much over a wide range of RHs (Supplementary Fig. 6a). This change in *V*_io_ primarily came from the change of the internal resistance *R*_s_ in the protein nanowires (as *V*_io_ = *R*_L_/(*R*_L_ + *R*_s_)·*V*_o_), which was highly sensitive to humidity (Supplementary Fig. 6b). As a result, this sensory circuit functioned as an active humidity sensor.

This active humidity sensor was connected to an artificial neuron made from a protein nanowire memristor (*M*) and a capacitor *C*_m_, with *C*_m_ emulating the membrane capacitance in a biological cell (Fig. 2b)^17,30^. On dry skin (RH < 50%), the sensor had a low output and only charged to a low membrane potential (e.g., *V*_m_ < 10 mV), with the memristor remaining Off (Supplementary Fig. 7). When the skin turned from dry to sweating (RH~90%) after exercise, the increased *V*_io_ charged *C*_m_ to an increasing *V*_m_ (Fig. 2c, upper panel). At *V*_m_ ~ 40 mV, the memristor was turned On and *C*_m_ quickly discharged. Thus, the artificial neuron produced a current spike that mimicked the action potential in neuronal firing (Fig. 2c, bottom panel). Here the threshold membrane potential of 40 mV was slightly lower than the *V*_th_ in *I*–*V* sweeps (Fig. 1b), because the charging process to *C*_m_ yielded a longer incubation time. Longer programming time can slightly offset the switching voltage in diffusive memristors^26^. These results demonstrate that the interface can serve as an artificial afferent circuit (Fig. 2b), in which the sensory signal was locally processed and converted into action potential (e.g., by an interneuron) before sending to the central nervous system for upper-level decision^9–11^. In this case, the interface can be used to perceive humidity level that is relevant to environmental and physiological conditions (e.g., hydration, wound state)^19^ for an intelligent decision.

The vertical protein nanowire device can also serve as a continuous energy source to drive other sensory interfaces responding to different stimuli, transcending specific-stimulus-typed interfaces relying on the careful selection of sensors to achieve a signal match to memristors. When a passive pressure sensor^31^ was combined with the protein nanowire device to form an active tactile receptor (Fig. 2d and Supplementary Fig. 8), the resistance change in the pressure sensor was converted to an active voltage signal across *R*_L_ through the voltage divider (Fig. 2e). This led to charging in the membrane potential (Fig. 2f, top panel) and subsequent neuronal firing (bottom panel). In a similar manner, replacing the pressure sensor with a passive optical sensor yielded an afferent optical neural interface (Fig. 2g and Supplementary Fig. 9). Optical stimulations were converted to active voltage signals (Fig. 2h) that could drive the neuronal firing (Fig. 2i). These results demonstrate the versatility in achieving self-sustained, multifunctional neuromorphic interfaces that can perceive and process different stimuli. These interfaces, which are fully environmental-driven, are inherently different from previous systems that either required external powering or were restricted to a specific type of stimulus for the signal match to memristors^7–11,28,29^. Two factors underlie the difference: (1) humidity is ubiquitous and hence provides truly continuous powering; and (2) the bio-amplitude signal processing removes the fundamental barrier in a signal mismatch.

### Frequency-driven neuromorphic interface

**Fig. 3:**
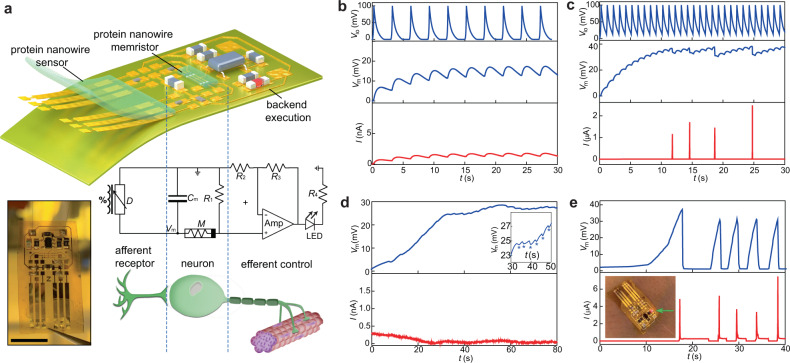
Integrated wearable neuromorphic interface. **a** Schematic (top) and circuit diagram (middle) of a fabricated (left) integrated wearable interface. Scale bar, 1 cm. *C*_m_ (10 µF), *R*_1_/*R*_2_/*R*_3_/*R*_4_ (20/1/100/1 kΩ), and an Op Amp (LM321) were used in the circuit. **b** Simulated evolution in the membrane potential (middle panel) and current (bottom panel) from the artificial neuron when receiving emulated normal (e.g., 0.3 Hz) respiratory sensing signal (top panel). **c** Simulated evolution in the membrane potential (middle panel) and current (bottom panel) from the artificial neuron when receiving emulated abnormal (e.g., 1 Hz) respiratory sensing signal (top panel). **d** Measured membrane potential (top panel) and current (bottom panel) from the artificial neuron with the sensor exposed to normal breathing. (Inset) The “stars” indicate discharging in membrane potential during breathing intervals. **e** Measured membrane potential (top panel) and current (bottom panel) from the artificial neuron with the sensor exposed to abnormal breathing. (Inset) The lighting of the LED (0805URC) triggered by neuronal firing.

We continued to explore functionality in the frequency domain, as physiological signals can be characterized by both amplitude and frequency (e.g., respiration). Diffusive memristors are promising candidates to emulate spiking bio-computation^32^. Here we implemented a skin-wearable integration and used respiration as the spiking stimulus. Figure 3a shows the schematic, diagram, and image of the wearable interface (see “Methods” section). A planar protein nanowire sensor that only responses to instant RH change was used to convert respiration into spiking signals (Fig. 1g, h). A compact sensor size of 0.3 cm^2^ was used to drive the bio-amplitude neuromorphic component. The sensor was connected to an artificial neuron comprised of a protein nanowire memristor (*M*) and a capacitor (*C*_m_). The capacitor modulates the membrane potential (*V*_m_) through the temporal integration of spiking inputs to trigger neuronal firing. Together, the sensor and artificial neuron form the front-end afferent circuit that processes the physiological signal for computation and decision. There can be different back-end circuits or efferent controls to execute the front-end decision. Here, an LED circuit was incorporated to visualize the neuronal firing, serving as a warning sign to abnormal respiratory rate.

To understand the dynamic response in the neuromorphic interface, we first constructed a memristor model to simulate the circuitry (Supplementary Fig. 10). With a low-frequency (e.g., 0.3 Hz) spiking input, emulating a normal breath rate (Fig. 3b, top), the charging to the capacitor associated with a breath is counteracted by discharging that takes place between intervals. As a result, the membrane potential is balanced at a low value of ~15 mV (Fig. 3b, middle), which is substantially below the *V*_th_ and does not trigger neuronal firing (Fig. 3b, bottom). With an emulated fast breath rate (e.g., 1 Hz, Fig. 3c, top), the shorter interval between breaths leads to reduced discharging and thus increased balancing membrane potential (Fig. 3c, middle). When the membrane potential approaches *V*_th_, it triggers neuronal firing. The neuronal firing quickly discharges the membrane potential to below *V*_th_, so that the neuron can continue to fire if the spiking inputs continue (Fig. 3c, bottom). Experimental results were consistent with these predictions from the simulation. At a normal breath rate (0.3 Hz), the measured membrane potential across the artificial neuron was below 30 mV (Fig. 3d, top), and the artificial neuron remained silent (Fig. 3d, bottom). However, when the breath rate was abnormal with an increased value of 1 Hz, the membrane potential increased to ~40 mV (Fig. 3e, top) and triggered neuronal firings (Fig. 3e, bottom). The neuronal firing can serve a decision signal to trigger back-end execution, which in this case was the lighting of an LED as a visual warning (inset, Fig. 3e).

This neuromorphic interface closely resembles a biological one, in which the sensory organelle and the processing neuron share the unitary signal (e.g., spiking action potential) that offers the efficiency for rapid decision^33^. Sensing and decision were accomplished without the need for an external power source. Pre-amplification circuit would be needed to drive an artificial neuron made from a typical memristor having 10× *V*_th_^26^, which would require external power (e.g., 100×) and increase integration complexity. Neither is desirable for future wearable systems or microsystems that place a high demand on compactness and energy sustainability.

### Reconfigurability

Biosystems adapt through reconfigurations. Self-healing electronics^34^ emulate system repair and physiologically transient electronics^35^ emulate system disintegration functions, but conventional electronics typically lack ready reconfigurability in both directions without substantial back-end resources (e.g., additional integrated circuitry) or a re-design of the integration. This limits the possibilities for tuning the response of neuromorphic interfaces to the dissimilar physiological signals (e.g., strength or frequency) of different conditions or people. In contrast, protein nanowire devices offer the possibility of simple reconfiguration because the protein nanowires in devices can readily be exchanged.

**Fig. 4:**
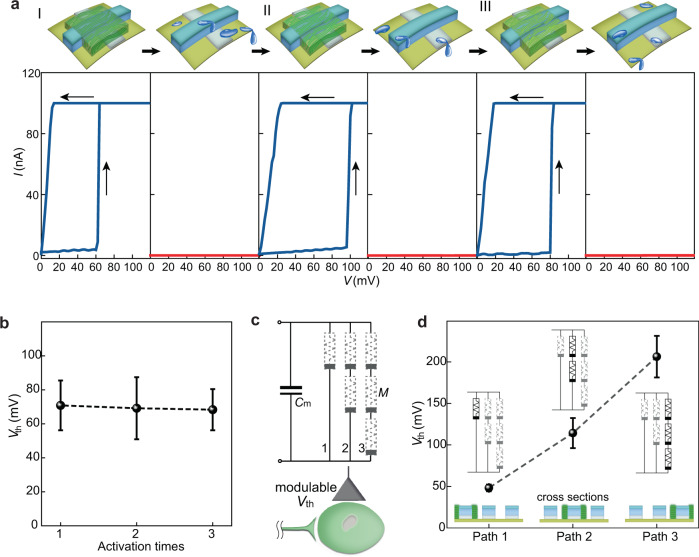
Reconfigurable neuromorphic interface. **a** (Top) Schematics of three consecutive processes of deposit-and-removal protein nanowires in a memristor structure. (Bottom) Corresponding memristive status in the device in each stage. **b** Average *V*_th_ (*N* = 6) in the three activated stages (i.e., deposited with protein nanowires). **c** Circuit diagram of a reconfigurable artificial neuron, with its threshold voltage (*V*_th_) for neuronal firing modulable by activating different paths of memristor arrays (*M*). **d** Measured *V*_th_ (*N* = 6) in the artificial neuron with different paths of memristor arrays activated. The error bars in the future represent the standard deviation (s.d.).

Individual protein nanowires retain structural integrity in a wide range of environments^16^. Thus, the films (Supplementary Fig. 1) formed from the molecular protein nanowires are stable in ambient environment^17–19,36^. The protein nanowires can be protonated or deprotonated in solution environments^37^, which can modulate the wire-wire interaction and hence film solubility. Protein nanowire film showed increasing solubility in water at higher pH (Supplementary Fig. 11) and thus memristors lost their bio-amplitude switching when the protein nanowire film was removed with a basic solution (Fig. 4a, I). Simple re-deposition of a new layer of protein nanowires on the same device, restored the bio-amplitude switching of the device (Fig. 4a, II). This process was repeated multiple times (Fig. 4a, I–III), without altering the bio-amplitude switching (Fig. 4b).

The presence or absence of protein nanowires can be used to tune artificial neuron response (e.g., for personalization). Six bare memristor structures (Ag–SiO_2_–Pt) were connected in combined series and parallel configurations (Fig. 4c). Depositing protein nanowires on the bare structure in line 1, formed an artificial neuron with a *V*_th_ of 50 mV (Fig. 4d, path 1). Dissolving protein nanowires in line 1 and depositing wires in line 2, yielded an artificial neuron with a *V*_th_ ~120 mV (Fig. 4d, path 2). Repeating the process to only activate line 3 increased *V*_th_ to ~200 mV (Fig. 4d, path 3). Protein nanowire sensors could be reconfigured by removing and replacing protein nanowires in a similar manner (Supplementary Fig. 12). Sensory output is also dependent on the protein nanowire thickness^18,19^, suggesting that system-level modulation of integration will be possible without complete device re-fabrication.

## Discussion

We have demonstrated fully self-sustained neuromorphic interfaces, which are based on a marriage between bio-amplitude signal processing and ubiquitous environmental energy harvesting, both enabled by the unique properties in protein nanowires. This marriage leads to comprehensive functionalities that are advantageous to those in existing neuromorphic interfaces (Supplementary Table 2). It also fundamentally closes the gap to biological integration that features a high level of intelligent signal processing and energy self-sustainability.

The protein nanowires, expressed on the exterior of G. sulfurreducens living in natural environments, are probably optimized for exceptional stability. Previously studies have shown that they are robust even against harsh conditions such as wide pH 2–10, detergents, organic solvents, and elevated temperature^16^. Latest studies show that thin-film devices made from them maintain the same device performance in energy harvesting and sensing beyond months in the ambient environment^18,36^. Oxidation in the exposed Ag electrode contributes to an extrinsic decay of stability in protein nanowire memristors, which can be addressed by adding a protective layer (e.g., Pt) to sustain the ambient stability (Supplementary Fig. 13). These results indicate that the protein nanowires may be readily employed for realistic applications. The fact that microbially produced protein nanowires are produced much more sustainably^15,16^ and are more readily modified for diverse functions^38^ than traditional electronic materials further add to the attractiveness. The “green” material composition offers advantages in biocompatibility and eco-friendliness for wearable and environmental applications. Other neuromorphic functions that work in different environments should be possible, generating a wide range of self-supported microsystems or intelligent sensors for broad deployments to support the Internet of Things.

Notably, the ability in constructed neuromorphic interfaces to function at low amplitudes is likely to facilitate direct electronic interfacing with biological signaling. The prospect of both biocompatibility and biostability in protein nanowire devices for tissue interfacing can be high due to the protein composition and exceptional stability discussed above, although future detailed studies are needed. Preliminary tests showed that protein nanowire memristor passivated with a thin layer of PDMS maintained the bio-amplitude function in a water environment (Supplementary Fig. 14). Therefore, device passivation may also be introduced to circumvent the direct exposure of protein nanowires to bodily fluids for implementation in tissue interfaces. Multidisciplinary efforts are also needed to explore the full potential of protein nanowires for expanding their functionalities^16^.

## Methods

### Synthesis of protein nanowires

The protein nanowires were harvested and purified from G. sulfurreducens as previously described^17–19^. Harvested nanowire preparation was dialyzed against deionized water to remove the buffer and stored at 4 °C. The resultant nanowire preparation yielded a measured pH ~9.

### Device fabrication

#### Protein nanowire memristors

Commercial Kapton polyimide (PI) film (thickness ~25 μm) was first cleaned by acetone, isopropyl alcohol, and deionized (DI) water. The bottom electrode (Ti/Pt, 3/17 nm) was defined by standard photolithography, metal deposition, and lift-off process. The dielectric layer and top electrode were defined together, by using standard photolithography, metal and dielectric depositions (Ti/SiO_2_/Ti/Ag, 3/20/2/150 nm), and lift-off process. Protein nanowire solution (50 µL/cm^2^, ~150 µg/mL) was drop-casted on defined device structures and thermally dried (80 °C, 1 min).

#### Protein nanowire sensors

The planar protein nanowire sensor (Fig. 1g) was fabricated following the procedure as previously described^19^. Briefly, a pair of interdigitated electrodes (Ti/Au 3/30 nm) was photolithographically defined on the PI substrate, followed by the drop-casting of protein nanowire solution. For the vertical protein nanowire sensor (Fig. 1d), Au-deposited PI films were defined by laser-cutting (GCC LaserPro Spirit GLS) to serve as the top and bottom electrodes. Protein nanowire film was deposited on the bottom electrode and covered by the top electrode. The device was sealed between two layers (~200 μm thick) of polydimethylsiloxane (PDMS, Sylgard 184, 10:1 mix ratio; Dow Corning) films to maintain the mechanical integrity, with an opening defined in the top PDMS layer (by laser-cutting) to allow the exposure of protein nanowire film to ambient humidity.

#### Integrated interface

After the definition of individual memristor and sensor structures following above procedures, a third interconnect layer (Ti/Au, 3/200 nm) was defined by standard photolithography, metal deposition, and lift-off process. Other circuit elements such as resistor and capacitor were pasted on defined pins using silver epoxy adhesive (MG Chemicals 8331). Protein nanowires were finally deposited.

### Characterizations

The electrical measurements were performed in the ambient environment, unless otherwise specified. The *I–V* curves were measured by using semiconductor parameter analyzers (Keysight B1500A and Agilent 4155C). The voltage and current outputs of protein nanowire sensors were measured by a source meter (Keithley 2401) and a semiconductor analyzer (Keysight B1500A). The relative humidity (RH) in the ambient environment was real-time monitored by a hygrometer (Model 8706; REED Instruments). A high-resolution scanning electron microscope (SEM, JSM-7001F; JEOL) was used to measure the device structures and film thickness. The nanowire networks were imaged by using a transmission electron microscope (TEM, JEM2200FS; JEOL).

## Supplementary information

Supplementary Information

## Data Availability

The data that support the findings of this study are available from the corresponding author upon reasonable request.
