# Supplementary material for: Self-sustained green neuromorphic interfaces
Source: Nat Commun. 2021 Jun 7;12:3351. doi: 10.1038/s41467-021-23744-2 (PMC8184933; doi:10.1038/s41467-021-23744-2)
Supplement: Supplementary file 1 — Supplementary Information [file 41467_2021_23744_MOESM1_ESM.pdf]

Supplementary Information

# Self-sustained Green Neuromorphic Interfaces

Tianda Fu[1], Xiaomeng Liu[1], Shuai Fu[1], Trevor Woodard[2], Hongyan Gao[1], Derek R. Lovley[2,3], Jun Yao[1,3,4]

1. Department of Electrical and Computer Engineering, University of Massachusetts, Amherst, MA, USA.
2. Department of Microbiology, University of Massachusetts, Amherst, MA, USA.
3. Institute for Applied Life Sciences (IALS), University of Massachusetts, Amherst, MA, USA.
4. Department of Biomedical Engineering, University of Massachusetts, Amherst, MA, USA.

**This PDF file includes:**

Supplementary Figures 1-14
Supplementary Tables 1-2
Supplementary References

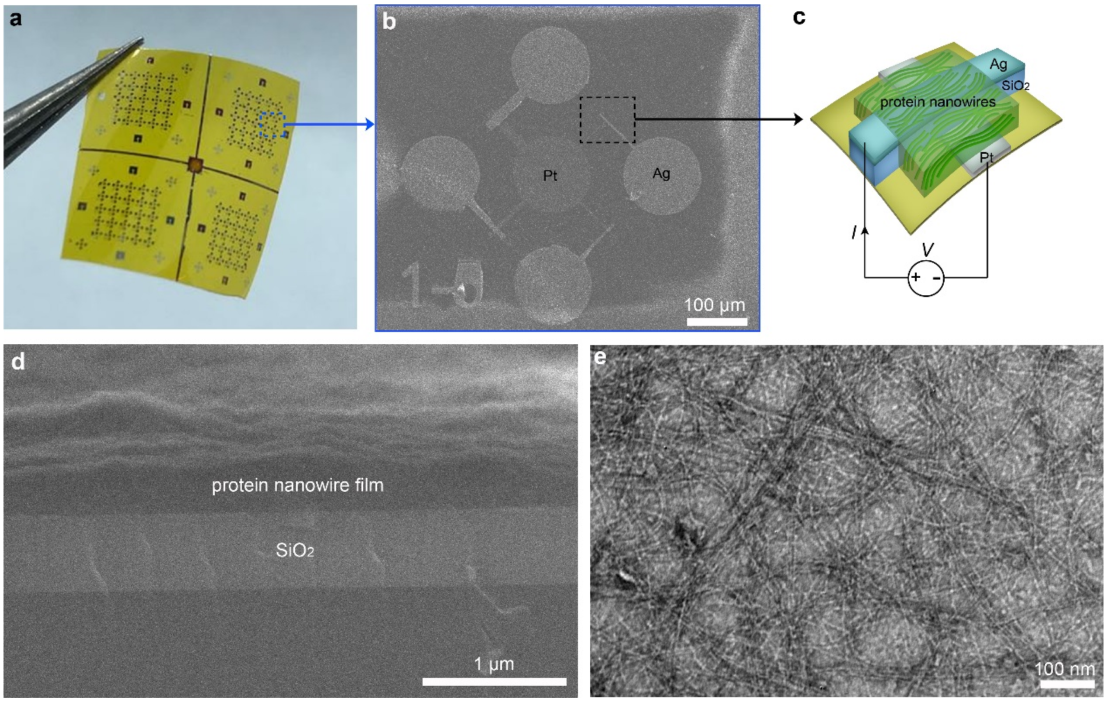


**Supplementary Figure 1 | Protein nanowire memristor structure**. (**a**) Optical image of fabricated protein nanowire memristor arrays on a flexible polyimide (PI) substrate. (**b**) SEM image of a cluster (4) of as-made protein nanowire memristors sharing the common bottom (Pt) electrode. Note that the nanowire film is less visible under SEM due to charging effect. (**c**) Schematic of the device structure and electrical testing setup. (**d**) Cross-sectional SEM image of a protein nanowire film deposited by drop-casting method. Note that a Si/$SiO_2$ substrate (instead of a polymeric substrate) was used for better imaging contrast. (**e**) TEM image of a thin layer of protein nanowires. Note that few-layer nanowires were used for imaging purpose. Here the nanowire density shown was not a close representation of the packing density in the actual film used in memristors (~500 nm thick) and in sensors (1-5 μm thick).

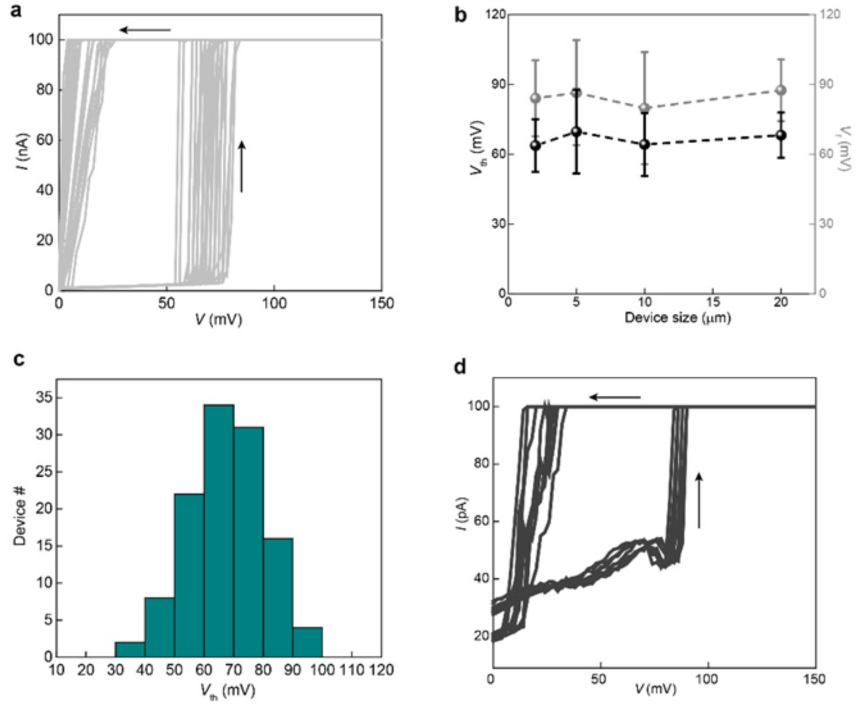


**Supplementary Figure 2 | Switching characteristics in protein nanowire memristors**. (**a**) 100 consecutive *I*-*V* sweeps (from 0 → 150 mV → 0) in a protein nanowire memristor, showing a narrow distribution of $V_{th}$ (69.2 ± 4.6 mV, ±s.d.). (**b**) Average switching voltage ($V_{th}$) and forming voltage ($V_f$) in protein nanowire memristors with respect to the change in device size (2 μm, 5 μm, 10 μm, 20 μm, *N*=30 for each size). The trends show that both $V_{th}$ and $V_f$ are largely independent of the device size, consistent with the filamentary switching mechanism.[1] (**c**) Statistics of $V_{th}$ from 117 protein nanowire memristors, showing a consistent distribution between 64.7±13.6 mV. (**d**) Bio-amplitude memristive switching in a protein nanowire memristor, achieving a programming compliance current as low as 100 pA (this device was fabricated on a $Si/SiO_2$ substrate with reduced Off current).

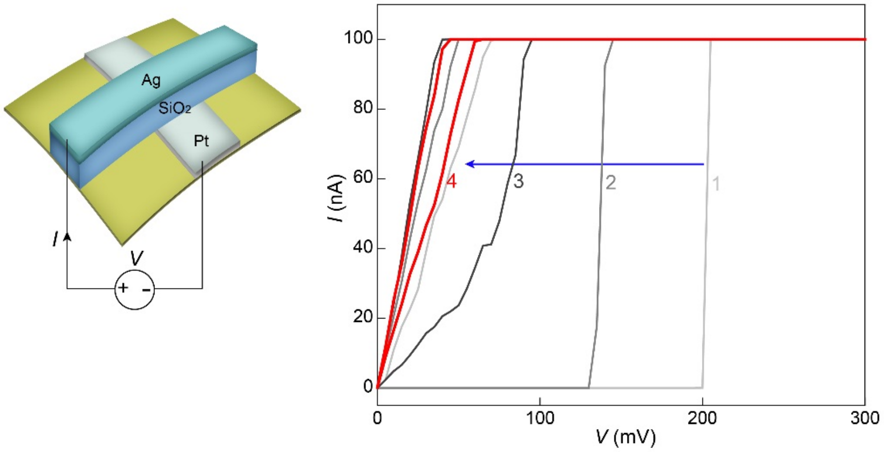


**Supplementary Figure 3 | Switching failure in bare Ag/$SiO_2$/Pt device without protein nanowires**. During attempted electroforming, the *I*-*V* sweeps in a bare Ag/$SiO_2$ (~20 nm)/Pt device structure (left schematic) show a trend toward switching failure (*e.g.*, forming non-switchable high-conductance state). The numbers indicate the *I*-*V* sweep order. All the tested devices (*N*=6) showed similar failure.

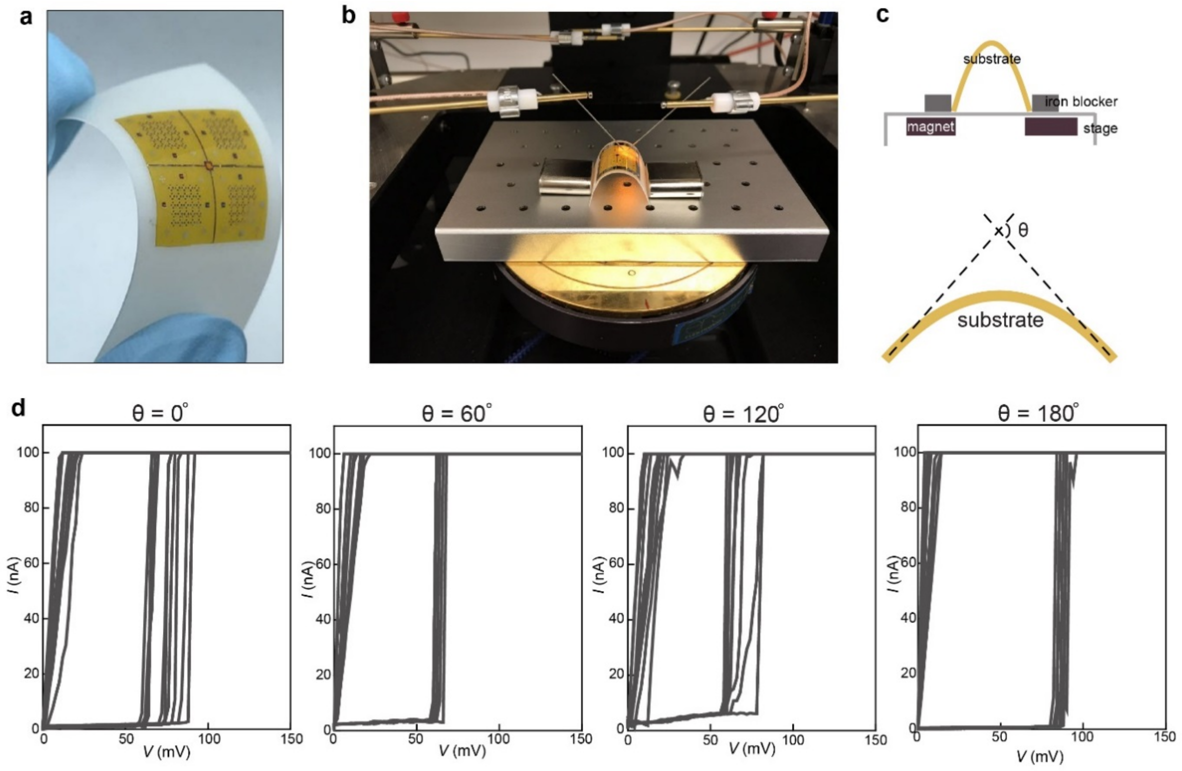


**Supplementary Fig. 4 | Bending test in memristors**. (**a**) To control the bending, the thin PI substrate (~ 25 µm thick) was further attached on a commercial polyethylene terephthalate (PET) substrate (~ 127 µm thick). (**b**) The experimental setup for testing the electrical performance of memristors in bending state. (**c**) (Top) schematic of controlling the bending state in the substrate using a home-built setup made from iron blockers, magnets, and an aluminum plate. (Bottom) schematic of the definition of a bending angle θ. The substrate had a lateral length ~2 cm. (**d**) Characteristic *I*-*V* curves (10 times each) from the same memristor at different bending angles of 0°, 60°, 120°, 180°, showing the maintenance of bio-amplitude memristive switching. Note that the repeated bending tests (Fig. 1c) were performed manually, with the bending angle close to 180°.

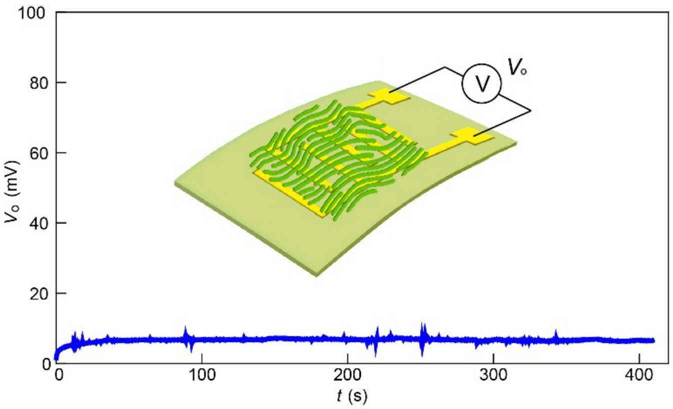


**Supplementary Figure 5 | Voltage output from a planar protein nanowire sensor**. The planar protein nanowire sensor only had a residual voltage output ($V_o$) of several millivolts in the ambient environment (*e.g.*, RH ~ 50%). This negligible output is consistent with the expectation of a lack of in-plane moisture gradient (and hence ionization gradient) due to structural symmetry. The residual voltage may result from some thickness variation in the protein nanowire film.

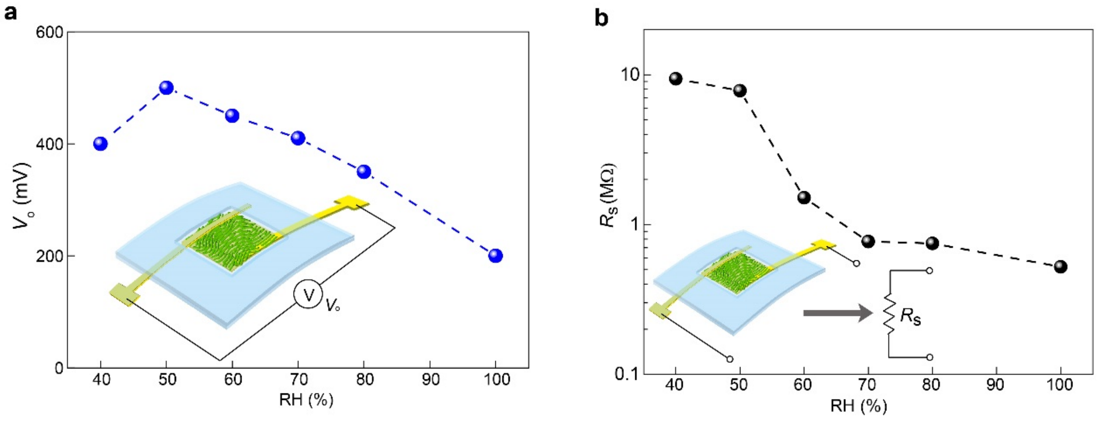


**Supplementary Figure 6 | Electrical characterizations in a vertical protein nanowire sensor**. (**a**) The open-circuit output voltage ($V_o$) maintained above 200 mV under different relative humidity (RH). The trend is consistent with previous study.[1] (**b**) Measured internal resistance ($R_s$) of the device under different RH. The trend shows that RH greatly affects $R_s$, which is also consistent with previous study.[2]

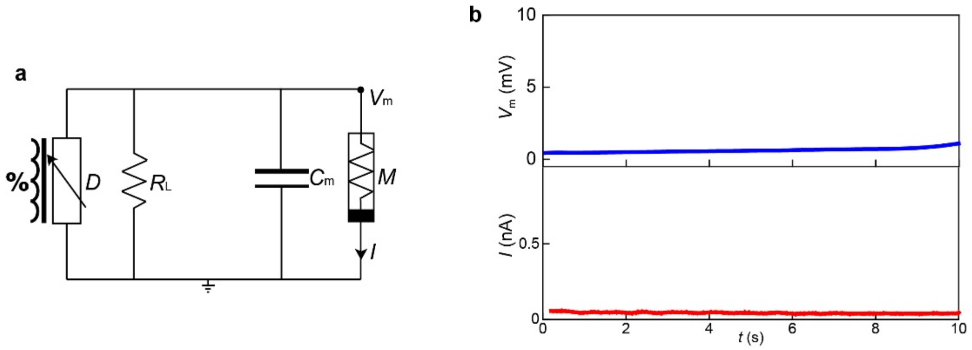


**Supplementary Figure 7 | Neuronal behavior with low input stimulus**. (**a**) Circuit diagram (same as Fig. 2b) of connecting a vertical protein nanowire sensor (*D*) to an artificial neuron made from a protein nanowire memristor (*M*) and a capacitor ($C_m$). (**b**) At a low relative humidity (*e.g.*, RH < 50%), the membrane potential $V_m$ in the neuron was only charged to a low value (*e.g.*, $V_m$ < 10 mV) due to the low output from the sensor (Fig. 2a), and the neuron remained silent without current spike (bottom).

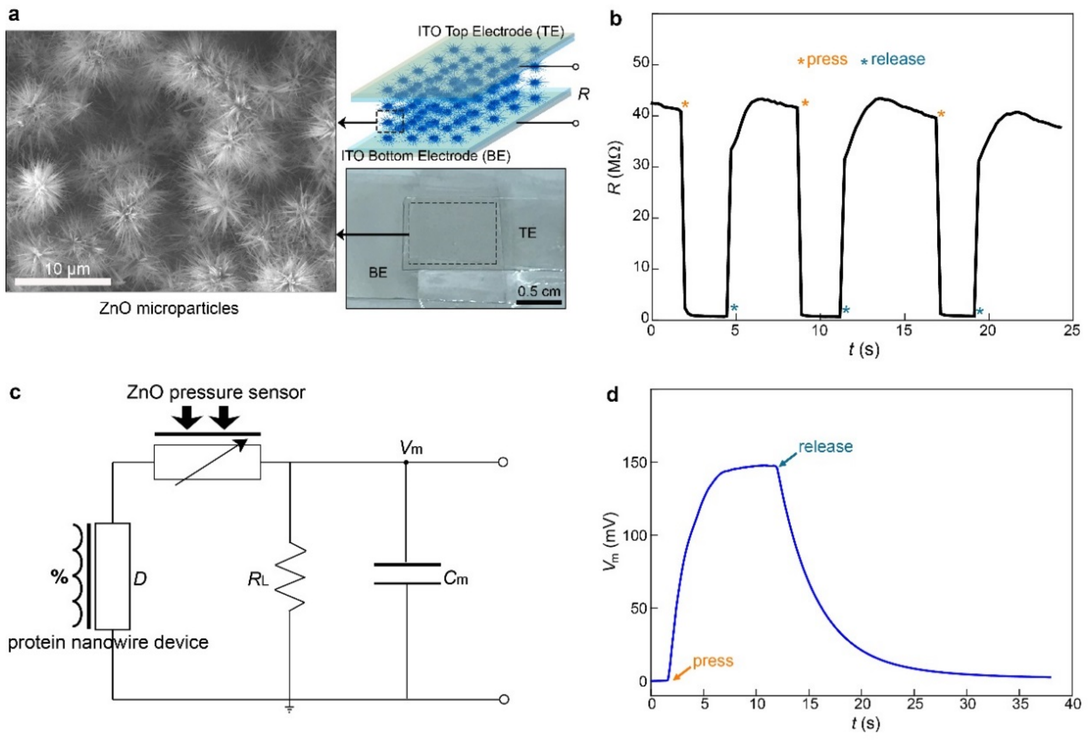


**Supplementary Figure 8 | Tactile receptor**. (**a**) Schematic (right top) and actual image (right bottom) of the resistive pressure sensor made by sandwiching a layer of bristled ZnO microparticles between a pair of ITO electrodes.[3] (Left) SEM image of the synthetic ZnO microparticles. The high-density 'nanospine' structures in the ZnO microparticles make the device highly sensitive to pressure. Details in the material synthesis and device fabrication can be found in previous study.[3] (**b**) Resistance change in the fabricated ZnO pressure sensor responding to repeated applied pressure (~10 kPa). (**c**) Circuit diagram of connecting the tactile sensory component to a (emulated membrane) capacitor ($C_m$ = 1 μF). The tactile sensory component was made from a vertical protein nanowire device (*D*, Fig. 1d) connected with the ZnO pressure sensor and a load resistor ($R_L$ = 2 MΩ). The protein nanowire device constantly harvested energy from the ambient humidity[1] and served as a powering source. The pressure sensor and $R_L$ formed a voltage divider. Pressure-induced resistance decrease in the pressure sensor will increase the voltage drop across $R_L$, which can charge the capacitor to a higher potential ($V_m$). (**d**) Representative evolution in $V_m$ modulated by pressing and releasing the pressure on the pressure sensor. The measurements were done in the ambient environment with a RH ~90%.

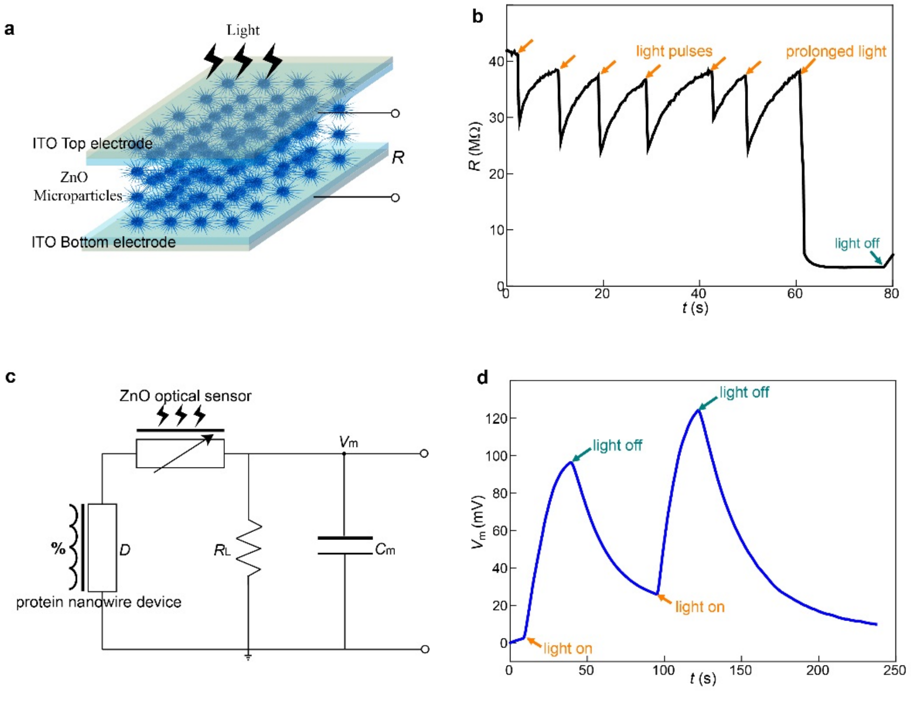


**Supplementary Figure 9 | Optical receptor**. (**a**) The optical sensor employed the same device structure (Supplementary Fig. 8a) by sandwiching a layer of ZnO microparticles between a pair of transparent ITO electrodes. Since the conductivity in ZnO is highly sensitive to UV light,[4] the resistance of the device can be modulated by UV illumination. (**b**) Resistance change in the optical sensor responding to pulsed (~0.5 s) and prolonged UV (395 nm) illuminations. (**c**) Circuit diagram of connecting an optical sensory component to a (emulated membrane) capacitor ($C_m$ = 1 μF). The optical sensory component was made from a vertical protein nanowire device (*D*) connected with the ZnO optical sensor and a load resistor ($R_L$ = 2 MΩ). The protein nanowire device constantly harvested energy from the ambient humidity and served as a powering source. The optical sensor and $R_L$ formed a voltage divider. Light-induced resistance decrease in the optical sensor will increase the voltage drop across $R_L$, which can charge the capacitor to a higher potential ($V_m$) (**d**) Representative evolution in $V_m$ modulated by tuning On and Off the illuminating UV light to the optical sensor. The measurements were done in the ambient environment with a RH ~90%.

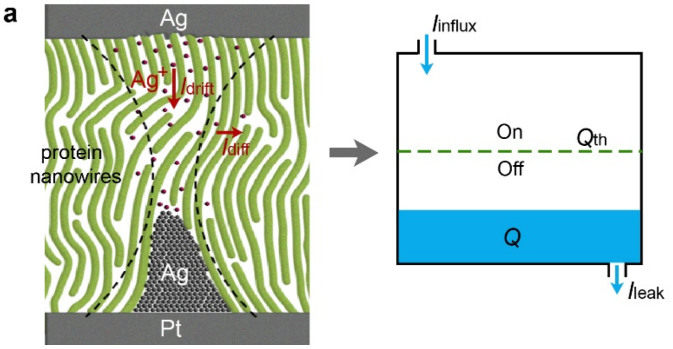


**Supplementary Figure 10 | Memristor modeling for circuit simulation**. (**a**) Previous analysis establishes that the dynamics of filament formation in the memristor can be treated with a leaky model.[5] Specifically, the filament formation is considered a threshold event when the net Ag number ($N$) reaches to certain value in a given filamentary volume. The Ag directly comes from $Ag^+$, which consists of a drift (influx) component $I_{drift}$ and a diffusive (leaky) component $I_{diff}$. Therefore, the Ag number can be related as $\Delta N \sim (I_{drift} - I_{diff}) \cdot \Delta t$. We assume that, before the filament formation, the $Ag^+$ ionic currents are directly correlated to the injection current. The filamentary model can then be further approximated to the model of a leaky charge reservoir (right schematic), in which the accumulative charge $Q(t)$ can be expressed as:

$$Q(t) = \int (V_{in}(t) \cdot G_{Off} - I_{leak}) \cdot dt \qquad (1),$$

where $V_{in}(t)$ is the instant applied voltage, $G_{Off}$ the Off conductance, and $I_{leak}$ the leaky current. Here we consider $I_{leak}$ to be constant, which is equivalent to the switching current obtained from experimental data ($I_{leak}$=$V_{th}$·$G_{Off}$, with $V_{th}$ ~35 mV and $G_{Off}$ ~$10^{-8}$ S). The threshold charge $Q_{th}$ (above which memristive switching is triggered) is also obtained from experimental pulse measurements as:

$$Q_{th} = (V_{pulse} \cdot G_{Off} - I_{leak}) \cdot \tau \qquad (2),$$

where $V_{pulse}$ is the amplitude of input pulse (*e.g.*, 100 mV), and $\tau$ the incubation time (*e.g.*, 10 ms) before the memristor turns on. Based on E.q. (1) and (2), we further define a sigmoid function $f(t)$ to fit the sudden transition around the switching threshold in the memristor as:[6]

$$f(t) = \frac{1}{1+e^{\frac{Q_{th}-Q(t)}{S}}} + \frac{1}{1+e^{\frac{Q_{th}+Q(t)}{S}}} \qquad (3),$$

where $S$ is a parameter defines the sharpness in transition. Here, we take $S = Q_{th}/100$, which is an empirical value obtained from comparing the fitting curve to the actual switching curve in the memristor.

Together, the output current $I(t)$ from the memristor can be expressed as the sum of the Off and On components:

$$I(t) = V_{in}(t) \cdot G_{Off} + V_{in}(t) \cdot G_{On} \cdot f(t) \qquad (4),$$

where $G_{On}$ ($10^{-3}$ $S$) is the On conductance of the memristor.[1]

(**b**) Circuit diagram used for simulating the dynamics in the artificial neuron ($C_m$=10 µF) in response to breath. The protein nanowire device is modeled by an ideal voltage source connected with a (internal) resistor ($R_S$ = 600 kΩ). The modeling and simulation were done using MATLAB 2019b and LTspice XVII.

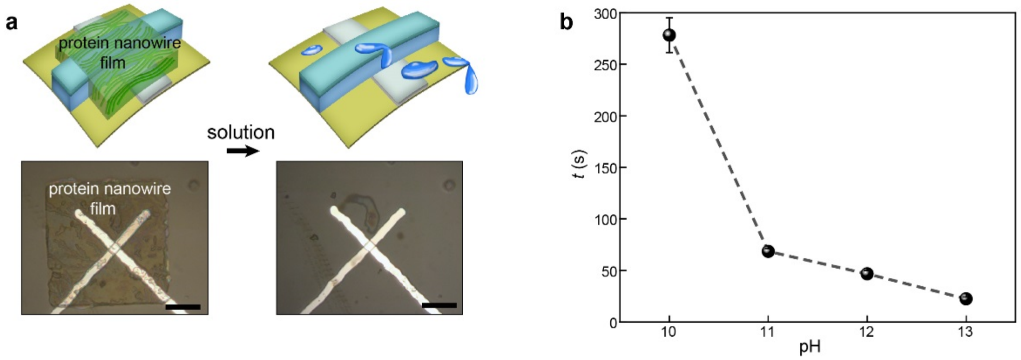


**Supplementary Figure 11 | Dissolving protein nanowires**. (**a**) Schematic (top) and optical images (bottom) of a protein nanowire memristor before (left) and after (right) dissolving the protein nanowire film by water solution. Scale bars, 8 μm. (**b**) The dissolving time (*t*) was observed to be highly dependent on the pH value in the solution. The protein nanowire films showed negligible solubility (*e.g.*, *t* = 10 min) in close-to-neutral (pH ~ 8.5) and acidic (pH ~ 1) solutions.

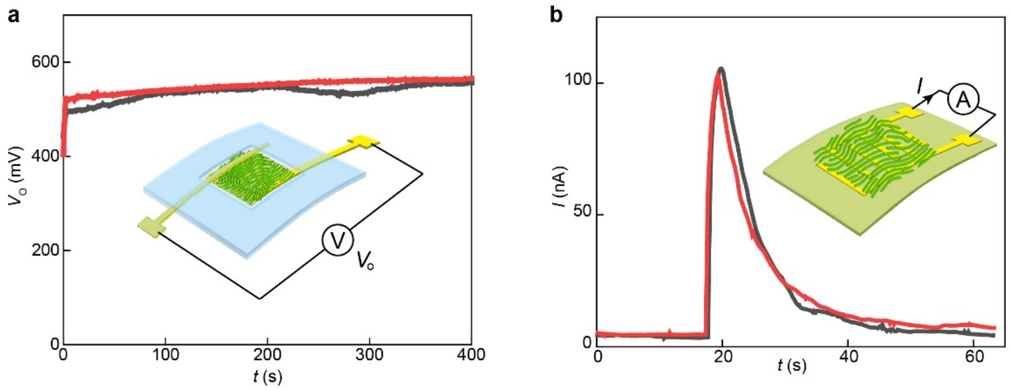


**Supplementary Figure 12 | Reconfigurable protein nanowire sensors**. Re-depositions of protein nanowires films in both (**a**) vertical and (**b**) planar nanowire devices yielded similar electric outputs (red curves) compared to outputs (gray curves) before the nanowire washout.

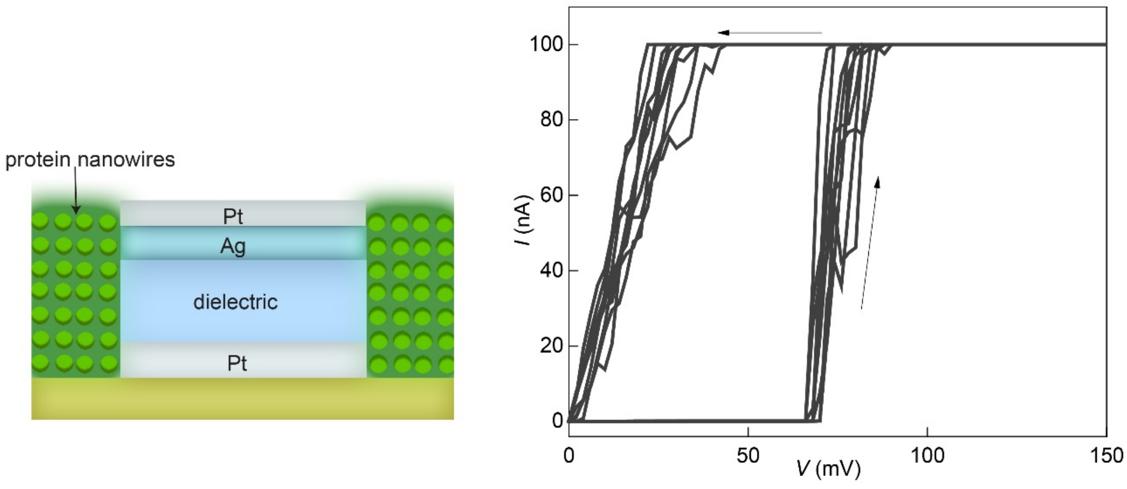


**Supplementary Figure 13 | Ambient stability in protein nanowire memristor**. Protein nanowire memristors (*N*=5), with the top Ag electrode passivated by an additional 10 nm Pt layer to prevent oxidation (left schematic), all maintained the bio-amplitude switching (right panel) in the ambient environment (*e.g.*, 14 days).

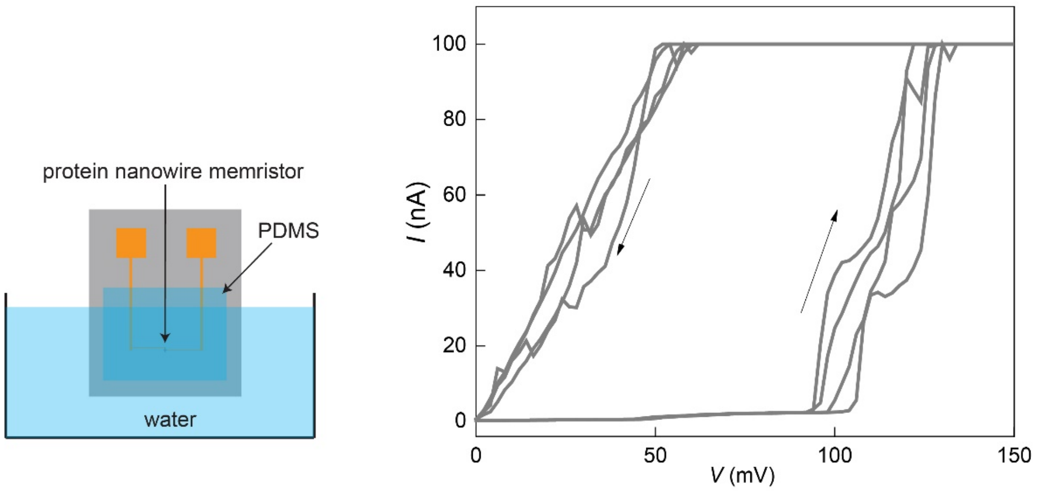


**Supplementary Figure 14 | Water resilience in memristor**. Fabricated protein nanowire memristor was dropcasted with polydimethylsiloxane (PDMS, Sylgard 184, 10:1 mix ratio) and cued in the ambient environment (RH~50%, 6.5 h). The PDMS-passivated memristor, when immersed in water (left schematic), maintained the bio-amplitude memristive switching (right panel).

**Supplementary Table 1**

Comparison of the average programming voltage ($V_{th}$), minimal programming compliance current ($I_{cc}$), and corresponding programming power ($V_{th}$×$I_{cc}$) in various similar type of (*e.g.*, volatile) memristors.[7]

| **Device structure** | **$V_{th}$ (V)** | **$I_{cc}$** | **Power** | **Ref.** |
|---|---|---|---|---|
| Pt/$TiO_2$/Ag | 40 | 1 nA | 40 nW | 8 |
| Pt/Vac/$Ag_2S$/Ag | 0.08 | 100 µA | 8 µW | 9 |
| Pt/Cu/$TaO_x$/Cu | 0.25 | 10 µA | 2.5 µW | 10 |
| Pt/α-$La_{0.3}Mn_{0.7}SrO_3$/Ag | 0.4 | 10 µA | 4 µW | 11 |
| Pt/$TiO_x$/Cu | 0.4 | 1 µA | 400 nW | 12 |
| Pt/$SiO_2$/Ag | 6 | 100 nA | 600 nW | 13 |
| Pt/$Ag_2S$/Ag/Pt | 0.45 | 100 nA | 45 nW | 14 |
| Pt/α-C/Cu | 0.55 | 50 µA | 27.5 µW | 15 |
| Cu/$HfO_2$:Cu/$HfO_2$/Pt | 0.4 | 10 µA | 4 µW | 16 |
| Pt/$SiO_2$/Cu | 0.6 | 500 µA | 300 µW | 17 |
| Pt/$SiO_2$/Ag | 0.2 | 10 µA | 2 µW | 18 |
| Pt/$ZrO_2$/Ag | 0.15 | 10 µA | 1.50 µW | 19 |
| Pt/MgO:Ag/Pt | 0.3 | 1 µA | 300 nW | 20 |
| Pt/$TiO_2$/TiN/AgTe | 0.4 | 100 µA | 40 µW | 21 |
| $p^+$-Si/$SiO_2$/$HfO_2$/Ag | 1.5 | 1 µA | 1.5 µW | 22 |
| W/$Cu_2S$/W | 0.3 | 10 µA | 3 µW | 23 |
| Pt/ferritin/Pt | 0.7 | 10 nA | 7 nW | 24 |
| Pt/ ZnO:Ag/Pt | 0.5 | 100 µA | 50 µW | 25 |
| Ti/h-BN/Cu | 0.3 | 100 µA | 30 µW | 26 |
| Pt/$HfO_2$/Ag | 0.3 | 10 µA | 3 µW | 27 |
| Pt/Ag/$SiO_2$:Ag/Pt | 0.5 | 10 nA | 5 nW | 28 |
| Pt/ZnO:Ag/Ti/PET | 0.4 | 10 µA | 4 µW | 29 |
| Pt/Ti/$VO_2$/Ti/Pt | 1.2 | 1 mA | 1.2 mW | 30 |
| Al/TiN/Ag/$GeTe_8$/Ag/TiN/W | 2.4 | 10 mA | 24 mW | 31 |
| Au/Ti/CuO/Ti/Au | 2.8 | 300 nA | 840 nW | 32 |
| Pt/CoO/ITO | 2.4 | 5 mA | 12 mW | 33 |
| Ag/$CsPbI_3$/Ag | 0.08 | 1 nA | 80 pW | 34 |
| Ag/$MoS_2$/Au | 0.35 | 100 µA | 35 µW | 35 |
| Ag/protein nanowire/Pt | 0.05-0.06 | 0.1 nA | ~6 pW | This work & Ref. 5 |

**Supplementary Table 2**

Comparison of the functionalities in some representative neuromorphic sensory interfaces.

| Neuromorphic interfaces responding to | | Flexible & Integrated | No external powering | Rebuildable device/circuit | Reference |
|---|---|---|---|---|---|
| Single-stimulus | Multi-stimulus | | | | |
| Tactile (pressure) | | | √ | | 36 |
| Tactile (pressure) | | | | | 37 |
| Tactile (pressure) | | √ | | | 38 |
| Optical (visible light) | | | | | 39 |
| Optical (UV-light) | | √ | | | 40 |
| Optical (mixed color) | | | | | 41 |
| Thermal (temperature) | | | √ | | 42 |
| Optical (visible light) | | | | | 43 |
| Tactile (pressure) | | √ | | | 44 |
| Tactile (pressure) | | | | | 45 |
| Tactile (pressure) | | √ | | | 46 |
| Tactile (pressure) | | √ | | | 47 |
| Tactile (pressure) | | | √ | | 48 |
| Optical (LED light) | | | | | 49 |
| Humidity (moisture) | | | | | 50 |
| | Optical, tactile, humidity | √ | √ | √ | This work |

**Supplementary References**


1. Liu, X. *et al*. Power production from ambient humidity using protein nanowires. *Nature* **578**, 550–554 (2020).
2. Liu, X. *et al*. Multifunctional protein nanowire humidity sensors for green wearable electronics. *Adv. Electron. Mater*. **6**, 2000721 (2020).
3. Yin, B., Liu, X., Gao, H., Fu, T. & Yao, J. Bioinspired and bristled microparticles for ultrasensitive pressure and strain sensors. *Nat. Commun*. **9**, 5161 (2018).
4. Lao, C. S. *et al*. Giant enhancement in UV response of ZnO nanobelts by polymer surface-functionalization. *J. Am. Chem. Soc.* **129**, 12096–12097 (2007).
5. Fu, T. *et al*. Bioinspired bio-voltage memristors. *Nat. Commun*. **11**, 1861 (2020).
6. Georgiou, P. S., Yaliraki, S. N., Drakakis, E. M. & Barahona, M. Window functions and sigmoidal behaviour of memristive systems. *Int. J. Circ. Theor. App*. **44**, 1685-1696 (2016).
7. Wang, Z. *et al*. Threshold switching of Ag or Cu in dielectrics: materials, mechanism, and applications. *Adv. Funct. Mater*. **28**, 1704862 (2018).
8. Hsiung, C.-P. *et al*. Formation and instability of silver nanofilament in Ag-based programmable metallization cells. *ACS Nano* **4**, 5414-5420 (2010).
9. Ohno, T. *et al.* Short-term plasticity and long-term potentiation mimicked in single inorganic synapses. *Nat. Mater*. **10**, 591-595 (2011).
10. Liu, T., Verma, M., Kang, Y. & Orlowski, M. Volatile resistive switching in Cu/$TaO_x$/δ-Cu/Pt devices. *Appl. Phy. Lett.* **101**, 0073510 (2012).
11. Liu, D., Cheng, H., Wang, G., Zhu, X. & Wang, N. Diode-like volatile resistive switching properties in amorphous Sr-doped $LaMnO_3$ thin films under lower current compliance. *J. Appl. Phys.* **114**, 154906 (2013).
12. Woo, J. *et al.* Control of Cu conductive filament in complementary atom switch for cross-point selector device application. *IEEE Electron Dev. Lett*. **35**, 60-62 (2014).
13. Sun, H. *et al.* Direct observation of conversion between threshold switching and memory switching induced by conductive filament morphology. *Adv. Funct. Mater*. **24**, 5679-5686 (2014).
14. Barbera, S. L., Vuillaume, D. & Alibart, F. Filamentary switching: synaptic plasticity through device volatility. *ACS Nano* **9**, 941-949 (2015).
15. Zhao, X. *et al*. Nonvolatile/volatile behaviors and quantized conductance observed in resistive switching memory based on amorphous carbon. *Carbon* **91**, 38-44, (2015).
16. Luo, Q. *et al*. Cu BEOL compatible selector with high selectivity ($>10^7$), extremely low off-current (~pA) and high endurance ($>10^{10}$). *IEDM*, pp. 10.4.1-10.4.4, (2015).
17. Chen, W., Barnaby, H. J. & Kozicki, M. N. Volatile and non-volatile switching in Cu-$SiO_2$ programmable metallization cells. *IEEE Electron Dev. Lett*. **37**, 580-583 (2016).
18. Liu, D., Cheng, F., Peng, R. & Yin, Y. Two resistive switching behaviors in Ag/$SiO_2$/Pt memristors. *IEEE-NANO*, 651-654 (2016).
19. Du, G., Wang, C., Li H., Mao, Q. & Ji, Z. Bidirectional threshold switching characteristics in Ag/$ZrO_2$/Pt electrochemical metallization cells. *AIP Advances* **6**, 085316 (2016).
20. Wang, Z. *et al*. Memristors with diffusive dynamics as synaptic emulators for neuromorphic computing. *Nat. Mater*. **16**, 101-108 (2017).
21. Song, J. *et al*. Monolithic integration of AgTe/$TiO_2$ based threshold switching device with TiN liner for steep slope field-effect transistors. *IEDM*, pp. 25.3.1-25.3.4 (2016).
22. Shukla, N. *et al.* Ag/$HfO_2$ based threshold switch with extreme non-linearity for unipolar cross-point memory and steep-slope phase-FETs. *IEDM*, pp. 34.6.1-34.6.4 (2016).

23. Lim, S. *et al*. Excellent threshold switching device ($I_{off}$ ~ 1 pA) with atom-scale metal filament for steep slope (< 5 mV/dec), ultralow voltage ($V_{dd}$= 0.25 V) FET applications. *IEDM*, pp. 34.7.1-37.7.4 (2016).
24. Zhang, C. *et al*. Convertible resistive switching characteristics between memory switching and threshold switching in a single ferritin-based memristor. *Chem. Commun*. **52**, 4828-4831 (2016).
25. Han, U.-B., Lee, D. & Lee, J.-S. Reliable current changes with selectivity ratio above $10^9$ observed in lightly doped zinc oxide films. *NPG Asia Mater*. **9**, e351 (2017).
26. Pan, C. *et al*. Coexistence of grain-boundaries-assisted bipolar and threshold resistive switching in multilayer hexagonal boron nitride. *Adv. Funct. Mater*. **27**, 1604811 (2017).
27. Yoo, J., Park, J., Song, J., Lim, S. & Hwang, H. Field-induced nucleation in threshold switching characteristics of electrochemical metallization devices. *Appl. Phys. Lett.* **111**, 063109 (2017).
28. Jiang, H. et al. A novel true random number generator based on a stochastic diffusive memristor. *Nat. Commun*. **8**, 882 (2017).
29. Park, Y., Han, U. B., Kim, M. K. & Lee, J. S. Solution-processed flexible threshold switch devices. *Adv. Electron. Mater*. **4**, 1700521 (2018).
30. Yi, W. *et al*. Biological plausibility and stochasticity in scalable $VO_2$ active memristor neurons. *Nat. Commun.* **9**, 4661 (2018).
31. Zhang, S. *et al*. Breakthrough in high ON-state current based on Ag–$GeTe_8$ selectors. *J. Mater. Chem. C* **8**, 2517-2524 (2020).
32. Huang, C.-H., Matsuzaki, K. & Nomura, K. Threshold switching of non-stoichiometric CuO nanowire for selector application. *Appl. Phys. Lett*. **116**, 023503 (2020).
33. Saitoh, S. & Kinoshita, K. Oxide-based selector with trap-filling-controlled threshold switching. *Appl. Phys. Lett*. **116**, 112101 (2020).
34. Zhu, X., Wang, Q., Lu, W. D. Memristor networks for real-time neural activity analysis. *Nat. Commun*. **11**, 2439 (2020).
35. Dev, D. *et al*. 2D $MoS_2$-based threshold switching memristor for artificial neuron. *IEEE Electron Dev. Lett*. **41**, 936-939 (2020).
36. Kim, B.-Y. *et al*. Nanogenerator-induced synaptic plasticity and metaplasticity of bio-realistic artificial synapses. *NPG Asia Mater*. **9**, e381-e381 (2017).
37. Wan, C. *et al*. An artificial sensory neuron with tactile perceptual learning. *Adv. Mater*. 30, 1801291 (2018).
38. Kim, Y. *et al*. A bioinspired flexible organic artificial afferent nerve. *Science* **360**, 998-1003 (2018).
39. Lee, Y. *et al*. Stretchable organic optoelectronic sensorimotor synapse. *Sci. Adv*. **4**, eaat7387 (2018).
40. Chen, S., Lou, Z., Chen, D. & Shen, G. An artificial flexible visual memory system based on an UV-motivated memristor. *Adv. Mater*. **30**, 1705400 (2018).
41. Seo, S. *et al*. Artificial optic-neural synapse for colored and color-mixed pattern recognition. *Nat. Commun*. **9**, 5106 (2018).
42. Yoon, J. H. *et al*. An artificial nociceptor based on a diffusive memristor. *Nat. Commun*. **9**, 417 (2018).
43. Kwon, S. M. *et al.* Environment-adaptable artificial visual perception behaviors using a light-adjustable optoelectronic neuromorphic device array. *Adv. Mater*. **31**, 1906433 (2019).
44. Shim, H. *et al*. Stretchable elastic synaptic transistors for neurologically integrated soft engineering systems. *Sci. Adv*. **5**, eaax4961 (2019).
45. Zhang, C. *et al*. Bioinspired artificial sensory nerve based on nafion memristor. *Adv. Funct. Mater*. **29**, 1808783 (2019).

46. Chen, Y. *et al*. Piezotronic graphene artificial sensory synapse. *Adv. Funct. Mater*. **29**, 1900959 (2019).
47. He, K. *et al*. An artificial somatic reflex arc. *Adv. Mater*. **32**, 1905399 (2020).
48. Zhang, X. *et al*. An artificial spiking afferent nerve based on Mott memristors for neurorobotics. *Nat. Commun.* **11**, 51 (2020).
49. Tan, H. *et al*. Tactile sensory coding and learning with bio-inspired optoelectronic spiking afferent nerves. *Nat. Commun*. **11**, 1369 (2020).
50. Tao, Y. *et al*. Moisture-powered memristor with interfacial oxygen migration for power-free reading of multiple memory states. *Nano Energy* **71**, 104628 (2020).
